# Supplementary material for: The interactome of intact mitochondria by cross-linking mass spectrometry provides evidence for coexisting respiratory supercomplexes
Source: Mol Cell Proteomics. 2017 Dec 8;17(2):216–32. doi: 10.1074/mcp.RA117.000470 (PMC5795388; doi:10.1074/mcp.RA117.000470)
Supplement: Supplemental Data [file supp_RA117.000470_133922_0_supp_23978_fzffwf.zip › spectra_annotation/mito_DR_spectra_annotation/397-1-7-1-1-1.pdf]

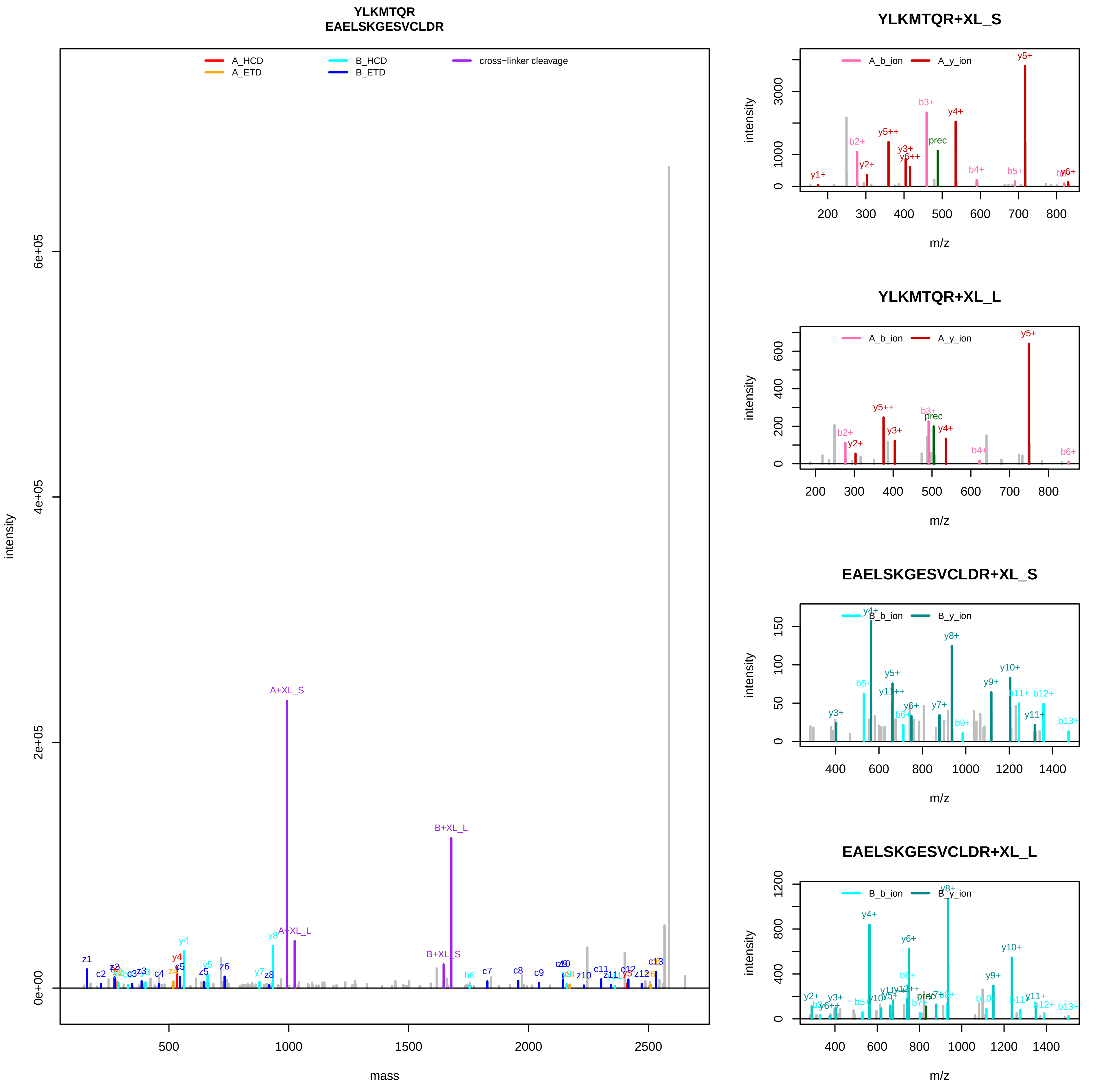
YLKMTQR
EAELSKGESVCLDR
A_HCD
A_ETD
B_HCD
B_ETD
cross−linker cleavage
intensity
mass
0e+00
2e+05
4e+05
6e+05
500
1000
1500
2000
2500
A+XL_S
B+XL_L
A+XL_L
B+XL_S
z1
c2
c3
z3
c4
y4
c5
y5
z5
z6
y7
y8
z8
b6
c7
c8
c9
z10
c11
c12
c13
y5
YLKMTQR+XL_S
YLKMTQR+XL_L
EAELSKGESVCLDR+XL_S
EAELSKGESVCLDR+XL_L
A_b_ion
A_y_ion
B_b_ion
B_y_ion
m/z
prec
y1+
y2+
y3+
y4+
y5+
y5++
y6+
y6++
b2+
b3+
b4+
b5+
b6+
y7+
y8+
y9+
y10+
y11+
y11++
b9+
b10+
b11+
b12+
b13+
